# Bibliography

**Published:** 1903-03-15

**Authors:** 


### BIBLIOGRAPHY.

The Consolidated Dental Manufacturing Company, has just published a small work by Dr W. H. Bruck, of Breslau, Germany, translated from the German by Dr. Charles W.

Jenkins, of Zurich, Germany. The books give a most comprehensive description of the technique of making porcelain inlays by the Jenkins system of low-fusing porcelain. By means of the many carefully prepared illustrations the text, which is very good indeed, is made very clear. There can be no question as to the value of this method of making inlays for certain cases. The results are beautiful, and the adaptation as perfect as may be desirable. The low fusing body makes it easy to manipulate, and this gives it a decided advantage over the higher-fusing bodies which require so much patience and skill to manipulate successfully, that few busy practitioners will give up the time required to produce proper results. This little book will enable any one to get an adequate knowledge of this method to make successful results certain and easy after careful reading and a little practice.

